# Squamous Cell Carcinoma in an Epidermoid Cyst

**Published:** 2013-04-26

**Authors:** Zachary J. Cappello, Morton L. Kasdan, Adam C. Augenstein, Saad P. Shaheen

**Affiliations:** ^a^Division of Plastic and Reconstructive Surgery, Department of Surgery, University of Louisville School of Medicine; ^b^Department of Pathology, Robley Rex VA Medical Center, Louisville, Ky

**Figure:**
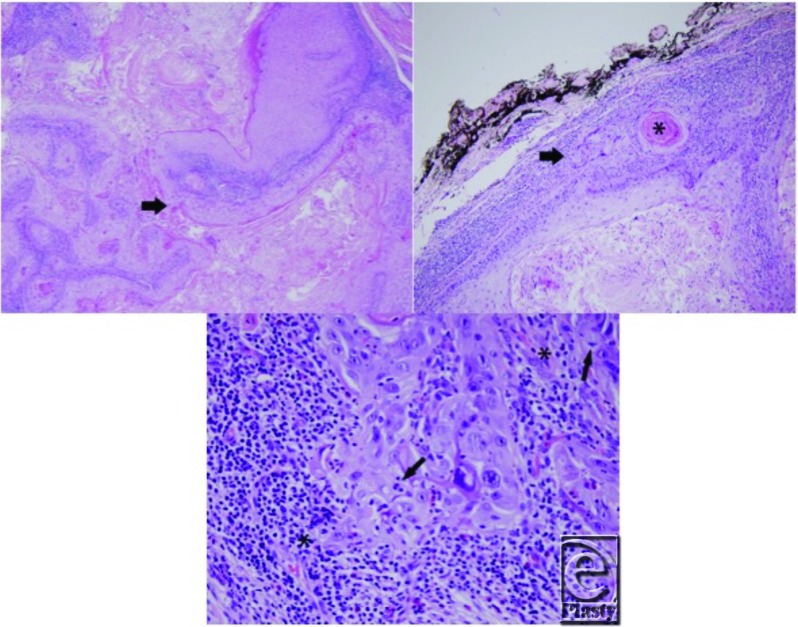


## DESCRIPTION

A 63-year-old white man presented with a raised, erythematous tumor on the left side of his nose. The lesion had been present for 3 years, and during the 6 to 8 weeks prior to his presentation, it had become painful. In addition, the cyst had started to drain purulent material when pressure was applied. On physical examination, the tumor was 20 mm in diameter and elevated 5 mm. There was a central punctum and was tender to palpation.

## QUESTIONS

1. **What is the differential diagnosis for the patient's lesion?**
2. **Is malignant transformation of an epidermoid inclusion cyst common?**
3. **What are potential exacerbating factors leading to malignant transformation?**
4. **What are the recommended studies after excisional biopsy of such a lesion?**

## DISCUSSION

The differential diagnosis for this patient's facial lesion included an epidermoid inclusion cyst, an abscess, cellulitis, and a lipoma. After excision, microscopy demonstrated a squamous epithelium-lined cyst with a granular layer (infundibular keratinization), laminated keratin, and keratinous debris. The cyst wall in the deep part of the cyst was thickened, but was benign. Originating from the acanthotic cystic wall is well-differentiated squamous cell carcinoma (SCC), showing keratin pearls and significant cytologic atypia. There was a clear-cut transitional zone between the benign cyst wall and the SCC, allowing distinction from cystic SCC, which shows malignancy with cystic degeneration. The squamous cell carcinoma was observed to extend to the peripheral and deep resection margins in relation to the deep dermal location of the cyst. The patient's clinical and histological findings were most consistent with an epidermoid cyst that had undergone malignant transformation.

Epidermal, or inclusion, cysts are frequently benign lesions lined with stratified squamous epithelium. Malignant transformation is an uncommon occurrence. Malignant transformation ranges from 0.011% to 0.045%.^1^^–^6 Anton-Badiola et al emphasized the need to distinguish a cyst with malignant change versus a cystic squamous carcinoma, in that the latter shows a direct connection and origin from epidermis as opposed to malignant change in an epidermal cyst, which is confined only to cyst wall. Eighteen well-documented cases exist in current literature. In 6 cases, the histological type was not recorded. Five cases presented cysts with histology demonstrating a well-differentiated squamous cell carcinoma. The remaining 7 cases were documented with a range of dysplastic changes.^1^^,^^3^ In this limited number of cases, only 3 presented with an aggressive course, in which metastasis and mortality developed within 5 to 10 months.^1^^,^^3^^,^^4^^,^^7^ Of these 3 cases with an aggressive course, the time to malignant transformation varied between 3 months and 28 years. The mean diameter of the reported cysts was 6.4 cm with a range of 5 to 9.2 cm. In 2 of the 3 cases, the cysts underwent malignant transformation to well-differentiated squamous cell carcinoma, while the remaining cyst underwent malignant transformation to poorly differentiated squamous cell carcinoma. Metastasis presented in a single lymph node in 2 cases while, in the remaining case, the cysts metastasized to the lung.

Chronic irritation and actinic damage have been proposed as exacerbating factors leading to malignant transformation of the existing lesions.^3^^,^^8^ The typical locations of such cysts, which include the head and neck (42.1% of cases), followed by the trunk and legs, support actinic injury pathogenesis.^1^^,^^3^ Chronic irritation as a cause of malignant transformation is supported by case reports describing long-standing lesions that developed malignant change. In addition, the potential repeated microtrauma or inflammatory nature of the cysts supports the claim that chronic irritation is a significant source of malignant transformation. Interestingly, one study examined 5 separate lesions for the presence of human papillomavirus as a potential pathogenetic mechanism from which malignant transformation could arise. While the results of this study yielded no conclusive evidence of the virus, it has been postulated that chronic irritation may exacerbate dysplastic process, similar to field effect, leading to malignancy.^9^ This is not documented in all cases; some display a distinct transition between the tumor and the wall of the cyst.^1^

Although uncommon, malignant transformation of the epithelium in an epidermoid inclusion cyst is a possibility in even benign-appearing lesions. Therefore, it is recommended that excisional biopsy of any such lesion should include histological examination for potential carcinomatous changes. If the margins are not clear of such carcinomatous changes, then reexcision with wide local margins must be undertaken. Fortunately, even when malignant change is encountered, metastasis is rare; thus, surgical excision with wide margins is usually sufficient.

## References

[1] López-Ríos F, Rodríguez Peralto JL, Castaño E, Benito A (1999). Squamous cell carcinoma arising in a cutaneous epidermal cyst. Case report and literature review. Am J Dermatopathol.

[2] Ziadi S, Trimeche M, Hammedi F (2010). Squamous cell carcinoma arising from an epidermal inclusion cyst: a case report. North Am J Med Sci.

[3] Antón-Badiola P, San Miguel-Fraile A, Peteiro-Cancelo Ortiz-Rey JA (2010). Squamous cell carcinoma arising on an epidermal inclusion cyst. Actas Dermosifiliogracias.

[4] Nemoto I, Shibaki A, Aoyagi S, Tsuji-Abe Y, Shimizu H (2006). Aggressive squamous cell carcinoma developing in a giant epidermal cyst of the abdomen. Int J Dermatol.

[5] Wheeton D, Strutton G, Weedon D, Strutton G (2002). Cysts, sinuses and pits: Appendageal cysts. Skin Pathology.

[6] Greer KE (1974). Epidermal inclusion cyst of the sole. Arch Dermatol.

[7] Jehle KS, Shakir AJ, ME Sayegh (2007). Squamous cell carcinoma arising in an epidermoid cyst. Br J Hosp Med.

[8] Chin-Yew L, Shyh-Chuan J (2002). Squamous cell carcinoma arising in an epidermal inclusion cyst. Chang Gung Med J.

[9] Morgan MB, Stevens GL, Somach S, Tannenbaum N (2001). Carcinoma arising in a epidermoid cyst: a case series and aetiological investigation of human papillomavirus. Br J Dermatopathol.

